# Improvement of "Bouncy Gait" in Lance-Adams Syndrome with Perampanel

**DOI:** 10.7759/cureus.6773

**Published:** 2020-01-25

**Authors:** Shen-Yang Lim, Dushyanth Babu Jasti, Ai Huey Tan

**Affiliations:** 1 Division of Neurology and the Mah Pooi Soo & Tan Chin Nam Centre for Parkinson’s & Related Disorders, Faculty of Medicine, University of Malaya, Kuala Lumpur, MYS; 2 Division of Neurology, Kasturba Medical College, Manipal, IND

**Keywords:** lance-adams syndrome, perampanel, myoclonus, negative myoclonus, bouncy gait

## Abstract

Lance-Adams syndrome (LAS) is chronic post-hypoxic myoclonus that is often associated with sudden lapses in muscle tone (negative myoclonus) in the legs, causing a disabling "bouncy gait." Given its relative rarity, there are no controlled treatment studies of LAS. The majority of cases require polypharmacy management, with an incomplete response. "Bouncy gait," in particular, is notoriously medication-refractory. Here, we report a patient with long-standing LAS who improved markedly when low-dose perampanel was added to his existing treatment regime consisting of clonazepam, levetiracetam, sodium valproate, and acetazolamide.

## Introduction

Lance-Adams syndrome (LAS) is chronic post-hypoxic myoclonus with onset days to weeks after a hypoxic episode, when the patient has regained consciousness [1]. The myoclonus is typically multifocal, worse with action, and associated with sudden lapses in muscle tone in the legs, causing a "bouncy gait" [1]. Additional features may include cognitive impairment, seizures, dysarthria, and ataxia [1]. Given its relative rarity, there are no controlled treatment studies of LAS. The majority of cases require polypharmacy management, with an incomplete response. "Bouncy gait," in particular, is notoriously medication-refractory [2]. Here, we report a patient with long-standing LAS who improved markedly when low-dose perampanel was added to his existing treatment regime.

## Case presentation

A 63-year-old Malaysian man of Chinese ethnicity underwent cardiac bypass surgery in 2013 for triple-vessel disease, which was complicated postoperatively by cardiac arrest resulting in hypoxic coma. He was hospitalized for three months, of which one-and-a-half months was in the intensive care unit. During his recovery, the patient experienced jerks involving the whole body and difficulty walking. There was also significant cognitive impairment. Epileptic seizures occurred during his hospitalization but not after discharge. He was treated with twice-daily clonazepam 0.5 mg, levetiracetam 500 mg, and sodium valproate 200 mg.

He was referred to our center in November 2017 from the rehabilitation physician with a diagnosis of "ataxia" causing gait difficulty as his main problem. On examination, there was an obvious "bouncy gait," requiring close supervision and, frequently, assistance to walk just several feet within the consulting room; gait was very hesitant and wide-based with an obvious tendency to falls. There was positive action myoclonus in the arms when performing the finger-to-nose test, more obvious on the left (mild-to-moderate amplitude) but no significant myoclonus at rest nor with the arms outstretched (and no asterixis). There was no significant stimulus-sensitivity to touch. Eye movements were normal, with mild-to-moderately slurred speech. There were no other features of cerebellar ataxia, Parkinsonism, or dystonia. The Montreal Cognitive Assessment (MoCA) score was 17/30. Brain MRI was normal. Lance-Adams syndrome (LAS) was diagnosed. Acetazolamide 250 mg bid was added, without significant improvement. We have in our patient medical records three video recordings taken, with the patient's written permission, over approximately two years, showing the relative stability of the patient's condition. These are available upon request from the corresponding author.

Due to ongoing gait difficulty and based on emerging reports of perampanel use in myoclonic disorders [3], we added perampanel in August 2019. Improvement occurred within the first week on 2 mg/d, with further improvement on 4 mg/d. A home video sent to us by the patient's family showed that his gait was obviously improved. He was able to walk independently, without a walking aid, at home and in a shopping center, with no "bouncy gait" evident. There were no adverse effects, and the benefit has been sustained at least until the time of writing of this report (October 2019).

## Discussion

Myoclonus in LAS is thought to be most commonly cortical in origin and may relate to abnormal gamma-aminobutyric acid (GABA) and serotonin neurotransmission in the brain [1]. There are no well-defined guidelines for treatment in LAS. Anti-epileptic drugs (AED) such as valproic acid, clonazepam, and/or piracetam/levetiracetam are commonly used, but treatment response is often unsatisfactory [1-2]; thus, there is need for new therapeutic options.

Perampanel is a recently introduced AED that antagonizes post-synaptic alpha-amino-3-hydroxy-5-methyl-4-isoxazolepropionic acid (AMPA) receptors. It is effective for seizures and myoclonus in different types of epilepsies, including progressive myoclonic epilepsies [3-4], with some patients regaining ambulatory capacity post-treatment [2]. Sedation, dizziness, anxiety, and irritability appear to be the most common adverse effects [3-4]. To our knowledge, there has been only one fully published case report [3] describing an improvement of myoclonus in LAS using perampanel (Table 1) [3,5-7]. A recent electrophysiological study reported that improvement in cortical myoclonus with perampanel correlated with improvements in the parameters of giant somatosensory-evoked potentials [7].

**Table 1:** Case reports of patients with Lance-Adams syndrome treated with perampanel ACET=Acetazolamide; CBZ=Carbamazepine; CLON=Clonazepam; F=Female; LAC=Lacosamide; LEV=Levetiracetam; M=Male; NA=Not Available; PER=Perampanel; PIR=Piracetam; PRIM=Primidone; UK=United Kingdom; VAL=Sodium valproate; ZNS=Zonisamide; 5-HT= 5-hydroxytryptophan

Variable	Steinhoff et al. Epilepsy Behav Case Rep 2016 (Germany) [3]	Lazaro Lopez et al. Eur J Hospital Pharmacy 2017 (Spain) (Abstract only) [5]	Yelden et al. Brain Injury 2019 (UK) (Abstract only) [6]	Oi et al. Clin Neurophysiol 2019 (Japan) [7]	Present Case Report (Malaysia)
Age of patient (years)	36	35	#1: 69; #2: 37	#1: 47; #2: 31	63
Gender	M	M	#1: M, #2: F	Both M	M
Antecedent event	Cardiac arrest due to Brugada syndrome	3 consecutive cardiac arrests	#1: Severe pneumonia; #2: Accidental decannulation of the tracheostomy tube	NA	Cardiac arrest in the postoperative period following cardiac surgery
Duration of LAS prior to PER (years)	1	NA	NA	NA	6
Medication treatment prior to PER (mg/d)	LEV (2000); VAL (1500); CLON (2); PIR (7,600); LAC (100), treatments were "in vain"	LEV; VAL; Propofol; Sodium thiopental; PIR; ZNS; Clonidine; Sodium oxybate; 5-HT; Gabapentin	LEV; VAL; CLON at "high" doses - patients said to be "resistant"	#1: LEV; CLON; PRIM; CBZ; PIR #2: CLON; PIR	LEV (1000); VAL (400); CLON (1); ACET (250)
Perampanel dose (mg/d)	2mg/d for 1^st^ 3 days, then 4mg/d	24	NA	#1: 10; #2: 4	2mg /d for 1^st^ week, then 4mg/d
Clinical response	Almost complete cessation of myoclonic jerks at 4mg, but no mention of gait improvement (wheelchair-bound)	"Controlled" the myoclonus	#1: Myoclonus greatly improved with improved function; #2: Function improved including ambulation and speech	#1: Myoclonus improved from marked to severe; #2: No improvement in myoclonus (remained moderate); Both improved in ADLs	
Drugs able to be reduced or discontinued	PIR and LAC stopped	All other medications besides LEV, gabapentin, PER, and risperidone were stopped	#1: CLON reduced; #2: CLON and VAL stopped	NA	Not yet
Follow-up period	>4 weeks	NA	NA	NA	6 weeks
Adverse effects	Somnolence, but this improved over time	"Behavioural disorders", requiring risperidone treatment	NA	#1: None; #2: Dizziness and palpitation	No

## Conclusions

In conclusion, we observed a remarkable improvement of LAS with low-dose perampanel treatment, which was also well-tolerated. This agent warrants further study in larger numbers of patients and over longer durations of follow-up, to confirm long-term efficacy and safety for treating LAS. All cases reported thus far have been treated with multiple medications before the introduction of perampanel, and further research should also determine if perampanel can be effectively employed earlier in the treatment course of LAS.

## References

[1] Gupta HV, Caviness JN (2016). Post-hypoxic myoclonus: current concepts, neurophysiology and treatment. Tremor Other Hyperkinet Mov (N Y).

[2] Stahl CM, Frucht SJ (2019). An update on myoclonus management. Expert Rev Neurother.

[3] Steinhoff B J, Bacher M, Kurth C, Staack AM, Kornmeier R (2016). Add-on perampanel in Lance Adams syndrome. Epilepsy Behav Case Rep.

[4] Canafoglia L, Barbella G, Ferlazzo E (2019). An Italian multicentre study of perampanel in progressive myoclonus epilepsies. Epilepsy Res.

[5] Lazaro López E, Jalon Urbina MM, Sánchez AP (2017). D1-096 Refractory Lance-Adams syndrome: pharmacotherapy management and iatrogenic complications. Eur J Hospital Pharmacy.

[6] Yelden K, Rendell L, Vardy L, Rose A, Crilly S, Merrison K (2019). Treatment of refractory myoclonus with perampanel in Lance Adams syndrome: a report of two cases. Accepted abstracts from the International Brain Injury Association's 13th World Congress on Brain Injury. Brain Inj.

[7] Oi K, Neshige S, Hitomi T (2019). Low-dose perampanel improves refractory cortical myoclonus by the dispersed and suppressed paroxysmal depolarization shifts in the sensorimotor cortex. Clin Neurophysiol.

